# The Potential Role of Intraoperative Ultrasonography in the Surgical Treatment of Hilar Cholangiocarci noma

**DOI:** 10.1155/1996/83272

**Published:** 1996

**Authors:** P. Watanapa, N. S. Hargrove, Y. Sirivatanauksorn

**Affiliations:** 1 Departments of Surgery and Radiology Siriraj Hospital Mahidol University Bangkok 10700 Thailand; 2 Faculty of Medicine Siriraj Hospital Mahidol University Bangkok 10700 Thailand

## Abstract

The role of intraoperative ultrasonography (IOU) in the surgical treatment of hilar cholangiocarcinoma was explored in twenty-two patients, 17 males and 5 females. The mean age was 55 years (range 36-78 years). Preoperative imaging studies included abdominal ultra-sonography and/or CT scan, and visceral angiography. Operations performed were segment III bypass in 18 patients, local resection of tumour in 2 and resection of tumour *en bloc* with left hepatectomy in 2. Interpretation of IOU in terms of vascular involvement by the tumour (as compared to angiography or operative findings) was correct in 21 patients; no vascular invasion in 20 and portal vein invasion in the remainder. One false negative result occurred in a patient whose IOU failed to show right hepatic artery encasement by the tumour. When compared to postoperative cholangiography or surgical specimen, IOU correctly demon-strated location and extent of the tumours in all but one patient who had incomplete tumour resection. IOU was also helpful in locating segment III duct for biliary bypass. The mean time used for IOU was 15.1 min (range 10-20 min.), and there was no procedure-related com-plication. When supplemented with operative exploration, IOU seems to be very useful in the assessment of the resectability of hilar cholangiocarcinoma.


*HPB Surgery*, 1996, Vol. 9, pp. 93–96
Reprints available directly from the publisher
Photocopying permitted by license only

© 1996 OPA (Overseas Publishers Association)
Amsterdam B.V. Published in The Netherlands
by Harwood Academic Publishers GmbH
Printed in Malaysia

### The Potential Role of Intraoperative Ultrasonography in the Surgical Treatment of Hilar Cholangiocarcinoma

P. WATANAPA, * N. S. HARGROVE and Y. SIRIVATANAUKSORN

Departments of Surgery and Radiology* Faculty of Medicine, Siriraj Hospital,
Mahidol University, Bangkok 10700, Thailand

(*Received July 1, 1994*)

The role of intraoperative ultrasonography (IOU) in the surgical treatment of hilar cholangiocarcinoma was explored in twenty-two patients, 17 males and 5 females. The mean age was 55 years (range 36–78 years). Preoperative imaging studies included abdominal ultra-sonography and/or CT scan, and visceral angiography. Operations performed were segment III bypass in 18 patients, local resection of tumour in 2 and resection of tumour *en bloc* with left hepatectomy in 2. Interpretation of IOU in terms of vascular involvement by the tumour (as compared to angiography or operative findings) was correct in 21 patients; no vascular invasion in 20 and portal vein invasion in the remainder. One false negative result occurred in a patient whose IOU failed to show right hepatic artery encasement by the tumour. When compared to postoperative cholangiography or surgical specimen, IOU correctly demon-strated location and extent of the tumours in all but one patient who had incomplete tumour resection. IOU was also helpful in locating segment III duct for biliary bypass. The mean time used for IOU was 15.1 min (range 10–20 min.), and there was no procedure-related com-plication. When supplemented with operative exploration, IOU seems to be very useful in the assessment of the resectability of hilar cholangiocarcinoma.

KEY WORDS: Intraoperative ultrasonography hilar cholangiocarcinoma

### INTRODUCTION

Surgical treatment of carcinoma arising at the confluence of the right and left hepatic ducts (hilar cholangiocarcinoma) remains a complex problem. Tumour removal, either by local excision or in conjunction with hepatic parenchymal resection, or palliative surgical biliary decompression is complicated by the anatomical variations of the area, and sometimes by the atrophic-hypertrophic changes of the liver in response to obstruction of the biliary system. During the past decade, advances in diagnostic imaging studies have improved patient selection for resective procedure or palliative biliary bypass[1–3]. At present, preoperative assessment of the resectability of the tumour depends mainly on cholangiography and visceral angiography.

Intraoperative ultrasonography (IOU) is now established as one of the most accurate techniques for detecting and locating liver tumours[4,5]. IOU can also demonstrate spatial relationships between tumours and adjacent vascular structures, thus facilitating tumour resection[4]. However, its role in the surgical treatment of hilar cholangiocarcinoma has not been reported yet. The high expenses of preoperative imaging studies (which is important for the medical care in a developing country) and the low resectability of cholangiocarcinoma (particularly in patients with liver flukes) prompted us to evaluate the potential role of IOU in the assessment of the extent and the resectability of hilar cholangiocarcinoma. The results of

*Address for correspondence*: P. Watanapa, Department of Surgery, Siriraj Hospital, Bangkok 10700, Thailand.

IOU were compared to those of cholangiography or surgical resected specimen and visceral angiography.

### PATIENTS AND METHODS

### Patients and Preoperative Investigations

This prospective study involved twenty-two patients with histologically proven hilar cholangiocarcinoma treated during September 1992–August 1993. There were 17 males and 5 females and the mean age was 55 years (range 36–78 years). Twenty patients (91%) resided in endemic area of liver flukes (Opisthorchis viverrini). All patients were investigated preoperatively by abdominal ultrasonography and/or CT scan; 18 had ultrasonography alone, 2 had CT scan only and 2 patients underwent both examinations. All but two patients had visceral angiography. The two patients who received no angiographic studies had obvious evidences of ascites (clinical and ultrasound or CT scan) suggesting of carcinomatosis peritonei. They were therefore not investigated to assess the resectability of their tumours. These preoperative studies were kept unseen by the operator of the IOU until the completeness of ultrasonographic evaluation in the operating theatre.

### Sequence During Operation and Operative Procedures

After entering the peritoneal cavity, abdominal viscera were routinely explored. Before any dissection around the liver, an intraoperative ultrasound probe (Toshiba, model IOB-502F) was applied on the sur-face of the liver, starting at the left side of the falciform ligament and then moved to the right. This probe was connected to the ultrasound tomographic apparatus (Toshiba, model SAL-32B). After the completion of IOU, the results of the study were recorded in a pre-pared data sheet. Decision to do a resective procedure or palliative biliary bypass was mainly based on the operative findings (with or without trial dissection of the tumour) and the results of the preoperative investigations. Only in a few cases, was it assisted by the findings of IOU.

Tumour resection with or without concomittent hepatectomy was attempted whenever there was no evidence of metastatic disease. Basically, for those who had tumours suitable for resection, we used the strategies introduced by Bismuth *et al.* as a guideline for tumour removal[6] (i.e. local resection for those whose tumours involved first-order ducts of both lobes and hepatic lobectomy for those whose tumours involved first-order duct of one lobe and second-order duct of the other). For those with tumour unsuitable for resection (Table 1), the segment III duct was identified by dissection into the umbilical fissure of the liver and sometimes could be easily located by IOU (placing the ultrasound probe on the surface of the liver just left to the falciform ligament). A Roux-en-Y segment III hepaticojejunostomy was then performed over a stent. This stent was left in place for 1 week and was removed after a tube-cholangiography. All patients were followed up by being scheduled to attend the clinic regularly or were contacted by post.

**Table 1** Reasons for not doing resective procedures among 18 patients

| | *No. of patients* |
|---|---|
| Multiple lymph node metastases | 10 |
| Liver metastasis | 2° |
| Carcinomatosis peritonei with liver metastatis | 2* |
| Poor general condition | 2 |
| Portal vein invasion with liver metastasis | 1# |
| Ascites with cirrhosis | 1 |

° These 2 patients died 1.5 and 3 months after surgery.
* These 2 patients died 1.5 and 4 months after surgery.
### This 54-year-old man died 2 months after surgery.

To evaluate the accuracy of IOU in the assessment of vascular invasion and the extent of tumour involvement of the biliary tree, the results of IOU were compared to those of preoperative angiography and postoperative cholangiography via the stent (patients who underwent segment III-enteric bypass) or the pathological reports of the resected specimen (patients who underwent resection). The sensitivity and specificity rates were obtained by using a two-by-two table.

### RESULTS

Eighteen patients underwent palliative segment III bypass, 2 had local resection of the tumour and the remaining two underwent resection of the tumour *en bloc* with left hepatectomy (with or without caudate lobe removal). There were two hospital deaths; a 78-year-old man who underwent local resection of the tumour and died 3 weeks postoperatively due to severe sepsis secondary to pneumonitis and a 36-year-old man with carcinomatosis peritonei who underwent segment III bypass and died of multiorgan failure 6 weeks after surgery.

Comparing IOU with preoperative angiography, IOU accurately demonstrated left portal vein invasion by the tumour in one patient (Figure 1). This 54-yearold man also had multiple small metastatic nodules on the surface of the liver (missed by preoperative ultrasonography) and therefore underwent palliative segment III bypass. One false negative study occurred in a 41-year-old female whose IOU failed to show hepatic artery encasement by the tumour. The remain-der had a normal study, both IOU and angiography. One of them had tumour compression (not invasion) of the portal vein causing narrowing of the vein, and this was detected clearly by IOU (Figure 2). Therefore the sensitivity rate of IOU in determining vascular involvement was 100% and the specificity rate was 95%.

Regarding the extent of tumour involvement of the intrahepatic ductal system, IOU showed first-order duct involvement of both right and left hepatic ducts in two patients who underwent local excision of the tumour. Histological examination of the resected specimen revealed microscopic incomplete resection of the tumour in one of them. Another two patients had tumour involvement of first-order duct of the right

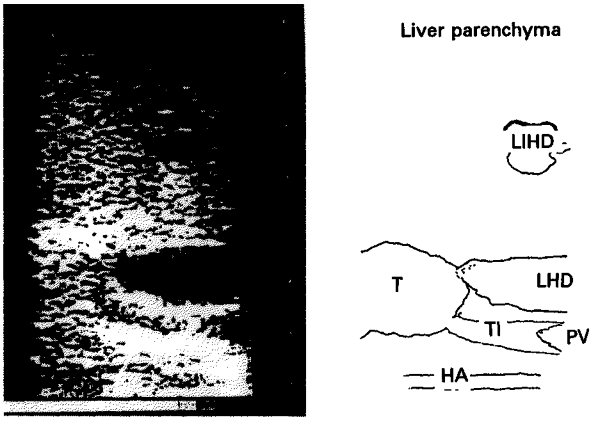

**Figure 1** IOU showing left portal vein invasion by tumour.

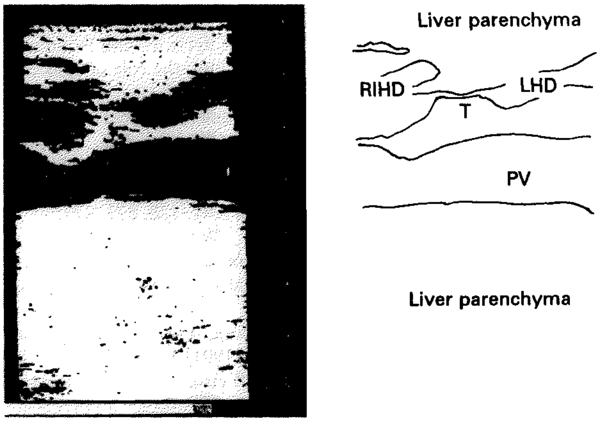

**Figure 2** IOU showing compression of portal vein by tumour.

hepatic duct and second-order duct of the left one and both underwent left lobe hepatectomy. Histological examination revealed complete removal of the tumours in both of them. Of the remaining 18 patients who underwent segment III bypass, IOU revealed second-order duct involvement of both lobes in 5 and second-order duct involvement of a single lobe in the remaining 13 cases. Resection was precluded in these 13 cases because of liver metastasis, carcinomatosis or poor general condition (see Table 1). IOU showed no communication between the right and the left ductal systems in 17 cases. Postoperative cholangiography via the anastomotic stent was performed in 16 of 18 patients with segment III bypass. In two patients, cholangiography could not be done because of tube displacement in one and the necessity of ventilatory support in the remainder (this patient died 6 weeks after the bypass). The cholangiogram corresponded well with the findings of IOU, particularly the biliary tree of the left lobe. For those with irresectable tumour and having atrophic-hypertrophic changes of the liver, IOU could demonstrate the segment III duct in all of them and helped to determine whether to approach the duct anteriorly or posteriorly in some cases. Time used for IOU varied between 10–20 minutes (mean = 15.1 minutes).

Postoperative complications occurred in 5 patients; 3 had upper gastrointestinal bleeding and 2 had cholangitis. All of them were treated successfully by conservative means. There were no IOU-related complications. During follow-up period, 10 patients died at 1.5–7 months after surgery (mean = 3.75 months). Six patients are still alive (5, 7, 8, 10, 13 and 15 months after operation). Four patients were lost to follow up.

### DISCUSSION

The prognosis of untreated carcinoma at the confluence of the hepatic ducts is grave, with a mean survival of less than 3 months[7]. During the past decade, several centres have reported improvements in survival of this cancer after aggressive surgical treatments[8–13]. Regarding the assessment of the resectability of the tumour, most surgeons still rely on preoperative CT scan, visceral angiography and cholangiography[8,11–13]. Intraoperative ultrasonography (IOU) has just emerged as a useful armament for surgery of the hepatobiliary system[4,5].

Both angiography and cholangiography carry significant risks to develop complications. In our centre, cholangiography for proximal bile duct obstruction is usually achieved by percutaneous puncture of the right lobe of the liver and followed by insertion of a catheter to decompress the biliary system. We do not have the facility of biliary endoprosthesis at present, therefore most patients with malignant obstructive jaundice have been treated by surgical biliary bypass. Although surgery is performed a few days after percutaneous biliary drainage, this approach still carries a high incidence of biliary tract infection particularly in those who underwent segment III bypass (right lobe cholangitis). Moreover, most patients with cholangiocarcinoma in Thailand, particularly those who reside in the endemic area of liver flukes, have Opisthorchis viverrini in their bile[14,15]. Though these patients may have resectable tumours on preoperative imaging studies, they are usually found to have irresectable lesions, peritoneal seedlings or small multiple liver metastases during operation. The lower resectability rate of hilar cholangiocarcinoma in this series (18%) as compared to that reported recently from other centres[8–11] can be explained, therefore, by the nature of the tumour and by the fact that most patients resided in the rural area of the country and sought medical advice only at a late stage of the disease.

Visceral angiography has been accepted as the best method to evaluate vascular invasion by malignancies particularly in cancer with a low resectability rate. Although the number of patients in this series is small, the high sensitivity and especially the specificity rates of IOU in demonstrating vascular involvement by tumours signified the potential role of using IOU to assess the resectability of hilar cholangiocarcinoma. Likewise, this study showed a possibility of using IOU to assess the extent of bile duct cancer. Although it is difficult to evaluate the accuracy of IOU in patients with segment III bypass, for those who underwent resective procedures, IOU findings corresponded well to the histopathology of the resected specimen.

Although the patients in this series had rather advanced diseases, the results disclose the potential benefit of using IOU in the surgery of hilar cholangiocarcinoma. We agree that visceral angiography and cholangiography are still the standard methods to assess the resectability of the tumour. However, in some centres particularly in the developing countries where such imaging studies are not available or difficult to obtain, combination of operative exploration, trial dissection of the tumour and IOU may help to determine the resectability of hilar cholangiocarcinoma. Moreover, IOU is also helpful in locating the segment III duct especially in patients with atrophic-hypertrophic changes of the liver.

### ACKNOWLEDGEMENTS

We thank the Faculty of Medicine Siriraj Hospital, Mahidol University, Bangkok, for supporting this research.

### REFERENCES

1. Gibson, R. N., Yeung, E., Thompson, J. N. *et al.* (1984) Clinicopathological aspects of high bile duct cancer. *Annals of Surgery*, **199**, 623–636.
2. Okuda, K., Ohoto, M. and Tsuchiya, Y. (1988) The role of ultrasound, percutaneous transhepatic chlolangiography, computed tomographic scanning, and magnetic resonance imaging in the preoperative assessment of the bile duct cancer. *World Journal of Surgery*, **12**, 18–26.
3. Choi, B. I., Lee, J. H., Han, M. C. *et al.* (1989) Hilar cholangiocarcinoma: comparative study with sonography and CT. *Radiology*, **172**, 689–692.
4. Choi, B. I., Han, J. K., Song, I. S., Kim, C., Han, M. C., Kim, S. T., Lee, H. S., Kim, C. Y. and Kim, Y. I. (1991) Intraoperative sonography of hepatocellular carcinoma: detection of lesions and validity in surgical resection. *Gastrointestinal Radiology*, **16**, 329–333.
5. Lygidakis, N. J. and Makuuchi, M (1991) Surgical anatomy of the liver in the presence of disease. Possibilities and expectations of perioperative ultrasonography. *Hepato-Gastroenterology*, **38**, 321–328.
6. Bismuth, H., Nakache, R. and Diamond, T. (1992) Management strategies in resection for hilar cholangiocarcinoma. *Annals of Surgery*, **215**, 31–38.
7. Kuwayti, K., Baggenstoss, A. H., Stauffer, M. H. and Priestly, J. T. (1957) Carcinoma of the major extrahepatic bile ducts exclusive of the papilla of vater. *Surgery Gynecology and Obstetrics*, **104**, 357–366.
8. Baer, H. U., Stain, S. C. Dennison, A. R. Eggers, B. and Blumgart, L. H. (1993) Improvements in survival by aggressive resections of hilar cholangiocarcinoma. *Annals of Surgery*, **217**, 20–27.
9. Tashiro S., Tsuji, T., Kanemitsu, K., Kamimoto, Y., Hiraoka, T. and Miyauchi, Y. (1993) Prolongation of survival for carcinoma at the hepatic duct confluence. *Surgery*, **113**, 270–278.
10. Ogura, Y., Mizumoto, R., Tabata, M., Matsuda, S. and Kusuda, T. (1993) Surgical treatment of carcinoma of the hepatic duct confluence: analysis of 55 resected carcinomas. *World Journal of Surgery*, **17**, 85–93.
11. Gazzaniga, G. M., Bagarolo, F. C., Ciferri, E. and Bondanza, G. (1993) Neoplasm of the hepatic hilum: the role of resection. *Hepato-Gastroenterology*, **40**, 244–248.
12. Childs, T. and Hart, M. (1993) Aggressive surgical therapy for Klatskin tumors. *American Journal of Surgery*, **165**, 554–557.
13. Nagorney, D. M., Donohue, J. H., Farnell, M. B., Schleck, C. and Iistrup, D. M. (1993) Outcomes after curative resections of cholangiocarcinoma. *Archives of Surgery*, **128**, 871–877.
14. Kurathong, S., Lerdverasirikul, P., Wongpaitoon, V. *et al.* (1985) Opisthorchis viverrini infection and cholangiocarcinoma: a prospective, case-controlled study. *Gastroenterology*, **89**, 151–156.
15. Srivatanakul, P., Parkin, D. M., Jiang, Y-Z, Khlat, M., Uaolans, U., Sontipong, S. and Wild, C. (1991) The role of infection by Opisthorchis viverrini, hepatitis B virus, and aflatoxin exposure in the etiology of liver cancer in Thailand: a correlation study. *Cancer*, **68**, 2411–2417.

